# Supplementary material for: Revealing core-valence interactions in solution with femtosecond X-ray pump X-ray probe spectroscopy
Source: Nat Commun. 2023 Jun 9;14:3384. doi: 10.1038/s41467-023-39165-2 (PMC10250333; doi:10.1038/s41467-023-39165-2)
Supplement: Supplementary file 1 — Supplementary Information [file 41467_2023_39165_MOESM1_ESM.pdf]

SUPPLEMENTARY INFORMATION

Revealing core-valence interactions in solution with femtosecond X-ray pump X-ray probe spectroscopy

Robert B. Weakly,[1] Chelsea E. Liekhus-Schmaltz,[1] Benjamin I. Poulter,[1] Elisa Biasin,[2, 3] Roberto Alonso-Mori,[4] Andrew Aquila,[4] Sébastien Boutet,[4] Franklin D. Fuller,[4] Phay J. Ho,[5] Thomas Kroll,[6] Caroline M. Loe,[1] Alberto Lutman,[4] Diling Zhu,[4] Uwe Bergmann,[7] Robert W. Schoenlein[2,4] Niranjan Govind,[3] and Munira Khalil[1,]*

[1]Department of Chemistry, University of Washington, Seattle, WA, 98195, USA

[2]Stanford PULSE Institute, SLAC National Accelerator Laboratory, Menlo Park, CA 94025, USA

[3]Physical and Computational Sciences Directorate, Pacific Northwest National Laboratory, Richland, WA 99352, USA

[4] Linac Coherent Light Source, SLAC National Accelerator Laboratory, Menlo Park, CA 94025, USA

[5]Chemical Sciences and Engineering Division, Argonne National Laboratory, Lemont, Illinois 60439, USA

[6] Stanford Synchrotron Radiation Light Source, SLAC National Accelerator Laboratory, Menlo Park, CA 94025, USA

[7]Department of Physics, University of Wisconsin-Madison, Madison, WI 53706, USA

[*]mkhalil@uw.edu

Contents

1 **Supplementary Note 1**: Estimation of signal strength in and out of the focal plane. 3

2 **Supplementary Note 2:** X-ray data filters 4

2.1 Bounds filters 5

2.2 Linearity filters 5

2.3 Pixel instance filter 6

3 **Supplementary Note 3**: Time-dependent valence occupancy 9

4 **Supplementary Note 4:** Additional X-ray spectra 9

5 **Supplementary Note 5**: Computational details 13

5.1 Electron cascade simulations 13

5.2 Electron structure calculations 14

Supplementary References 17

1 **Supplementary Note 1**: Estimation of signal strength in and out of the focal plane.

Optimal utilization of photons as a resource implies future experiments should effectively compare probe spectra before and after transmission on a shot-to-shot basis. Lacking an effective map between upstream and downstream spectrometers, we compare the measured probe transmission for in focus and out of focus conditions. In this experiment, the signal strength in a given volume is proportional to the product of the X-ray pump and X-ray probe field strength. The relationship for signal strength (*S*), and corresponding beam area (A) leads to the quartic relationship with displacement along the direction of travel (Δz = +2 mm), as seen in Supplementary Equation 1, below:

$$\frac{S_{out}}{S_{in}} = \frac{{A_{\text{in}}}^2}{{A_{\text{out}}}^2} = \left(1 + \frac{\theta_h \Delta z}{D_h} + \frac{(\theta_h \Delta z)^2}{{D_h}^2}\right)\left(1 + \frac{\theta_v \Delta z}{D_v} + \frac{(\theta_v \Delta z)^2}{{D_v}^2}\right). \quad (1)$$

In the above expression, D_h and D_v are the diameters of the focused X-ray beam in the horizontal and vertical dimensions. For the assumptions listed above, the change in the XPXP signal strength is shown in Supplementary Figure 1.

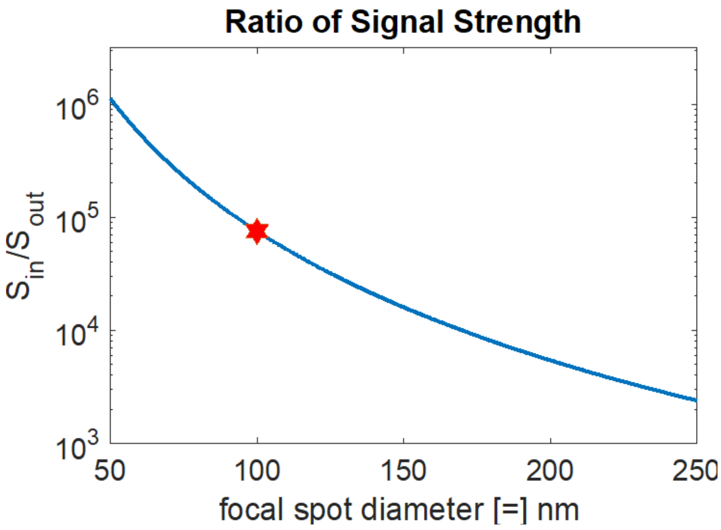

Supplementary Figure 1. Comparison of predicted signal strength for in and out of focus experimental conditions. Experimental measurements indicate an initial diameter of 100 nm (shown by red star). Error in the diameter of a factor of 2 would still result in a drop of signal strength greater than 10^3.

Two sets of Kirkpatrick-Baez (KB) mirrors are positioned to independently adjust the focal plane (z) in both the horizontal and vertical dimensions of the beam profile. The two mirrors, at different distances from the focus (0.9 and 0.5 m), impart an elliptical cone of convergence to form the focus (θ_h = 0.63, θ_v = 1.02 milliradians) in the horizontal and vertical dimensions respectively. Imprint etchings taken during the experiment indicate a focal diameter of 100 nm.[1]

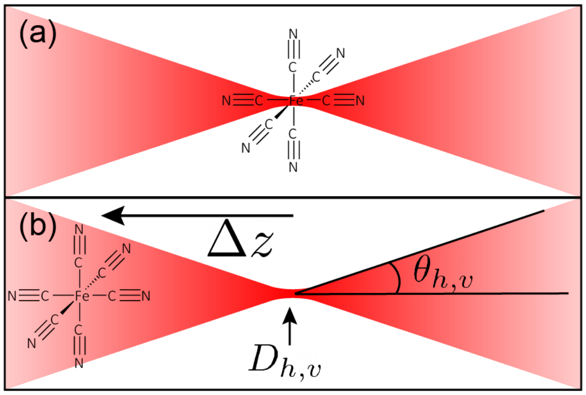

Supplementary Figure 2. Position of molecular system relative to the focus for the (a) pumped and (b) unpumped configurations. Δz, $D_{h,v}$, and $\theta_{h,v}$ are shown as defined in Supplementary Equation 1.

2 Supplementary Note 2: X-ray data filters

Supplementary Table 1. Total number of shots analyzed following bounds and linearity filters in each XPXP spectrum

Substance	Condition	Shots Pumped	Shots Unpumped	Figure
Fe^{II}	Full Fluence	165,835	174,422	3 (a)
Fe^{III}	Full Fluence	220,428	168,162	3 (b)
Solvent	Full Fluence	28,050	27,584	Supplementary Figure 3
Fe^{II}	Fe Foil Attenuated	88,351	29,715	Supplementary Figure 6

In the discussion below, "High" and "Low" refer to the high and low photon energy pulses, respectively. Two photodiodes (10 mm x 10 mm Hamamatsu Si PIN, Model: S3590, covered in visible light blocking black Kapton) collected scatter off the diamond chamber window. The diodes were positioned directly downstream of the KB mirrors, ~75 mm from the diamond

window, oriented away from the liquid jet. One diode included an additional 100 μm thick Fe foil to block the pump pulse and measure only the probe pulse intensity.

2.1 Bounds filters

Bounds filters apply two conditions (listed below as [$cond_{floor}$, $cond_{var}$]) based on a single self-normalized diode or metric such that the mean of the metric is one. All shots which are either lower in normalized metric value $cond_{floor}$ or deviate from the mean by $cond_{var}$ number of standard deviations, are eliminated.

i. (Low) Photon Intensity Diode (Upstream) [0.055, 2.3]

ii. (High) Photon intensity Diode (Upstream) [0.02, 2.2]

iii. (High) Photon Energy (Upstream) [0.945, 2.2]

iv. (Total) Intensity Diode (Upstream) [0.2, 2.2]

2.2 Linearity filters

These filters remove shots whose characteristics of expected linearity exceed the listed bounds for each diode or sensor by the listed fraction of the domain. If the data cover a span of 1,000 arbitrary units with a bound of 0.02, shots which deviate from the best fit line by more than 20 are removed.

i. X: (High) Photon Intensity Diode (Upstream)

Y: Fluorescence Diode (Downstream)

Bound: [0.5]

ii. X: (Low) Photon Intensity Diode (Upstream)

Y: (Low) Integrated Photon Energy Spectrum (Upstream)

Bound: [0.09]

2.3 Pixel instance filter

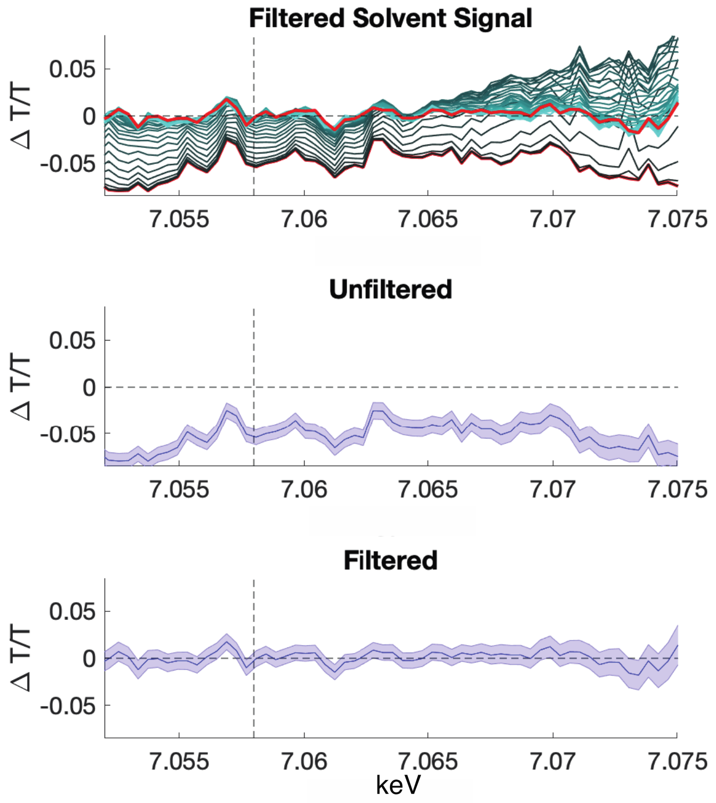

Supplementary Figure 3. TOP: Progressive effect of ΔT/T from pixel instance filter. Spectra correspond to evenly spaced thresholds between 0→1 in steps of 0.025. Lower red line corresponds to no filter applied. Upper red line corresponds to threshold = 0.78. MIDDLE: Solvent signal without filtering. BOTTOM: Solvent signal with final filter (threshold = 0.78). Total signal disappears. Vertical dashed lines represent the atomic Fe Kβ fluorescence line.

Two considerations in the spectral measurement of SASE pulses are: (1) Due to the photon-energy fluctuations from pulse-to-pulse, the ensemble spectral width (averaged over many pulses) exceeds the spectral width of an individual pulse, and (2) subsequent shots have little to no correlation in photon energy other than the same statistical average that all shots share in an ensemble. This creates a statistical illusion as the typical Gaussian profile indicates that each shot carries a small intensity in the wings. This is represented as a weak intensity measured at each shot. In reality, SASE pulses are intense regardless of their deviation from the average photon

energy. There are simply fewer shots with appreciable intensity in the wings and result in poorly-sampled high-intensity readings rather than well-sampled low-intensity readings.

Supplementary Figure 3 shows the effect of applying the pixel instance filter (PIF) on solvent data scaling the threshold from 0→1 and at the final threshold. Note that this removes the apparent offset signal of ~5% and removes the false bias showing a positive signal at hν>7.065 keV. A consequence of this data reduction is the increase in measurement uncertainty as discussed above. The elimination of uniform offset in sufficiently large data sets indicates the validity in considering the mean subtracted difference spectra for small data sets. To make this point, Supplementary Figure 6 shows data both with and without the mean subtracted. Threshold values were determined by an automated procedure, the rate of change in the difference signal was measured as a function of the threshold value and the smallest threshold with a negligible rate of change was selected.

Supplementary Figure 4 shows the effect of applying the PIF on approximately 70 randomly selected shots. Pixels with low intensity are removed from analysis. This dramatically reduces the number of points that are considered in analysis, but also restricts the analysis to the subset of shots in which photons interact with the detector. Each horizontal line in Supplementary Figure 4b shows an individual pulse spectrum. The upper portion of the plot includes all of the data, but most of the pixels are nearly zero. If we remove the lowest values from each spectrum (lower portion) we see that each shot leaves the majority of the energy bandwidth relatively untouched. If we were to simply take the value of each pixel (energy bin) as its raw readout value we would be misrepresenting the spectrum of the pulse. For this reason, we filter (remove from analysis) each instance of a pixel that fails to reach a minimum threshold intensity, which scales with the average pulse intensity. This can be viewed as eliminating the pixel instances which contain no chemical information and windowing each shot to the most data-rich pixels. For

instance, after the application of this filter, the pixel on the far right of Supplementary Figure 4b would only consider the 6 shots in the lower panel and 3 in the upper panel with appreciable intensity. Its uncertainty would increase, changing in proportionality from $\propto {}^{1}/_{\sqrt{70}}$ to $\propto {}^{1}/_{\sqrt{9}}$, but the 9 shots which are considered are the only 9 that carry any information. The increase in uncertainty is seen in panel Supplementary Figure 4a in which the red bounds of the PIF data are wider than those of the blue (raw) data. Further, the sloped shape of the spectrum disappears, depicting the wings of the spectrum as intense, but less well sampled.

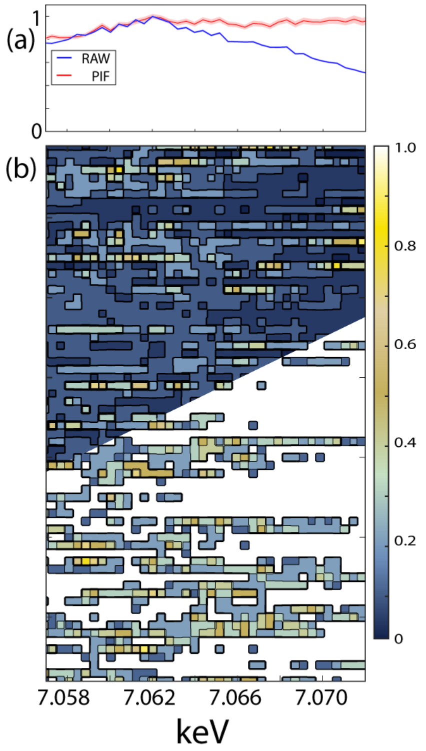

Supplementary Figure 4. (a) Average probe spectrum pre (blue) and post (red) application of the pixel instance filter (PIF) over a randomly selected 1,000 shots detected through solvent only. Shaded areas represent standard error. **(b)** Sample of initial shots in the subset averaged in (a). Each row represents a single shot. The upper region is unfiltered data with small contributions (close to zero) in all shots. The lower region represents data following filter threshold applied. For a given vertical slice (pixel) there are fewer considered shots, increasing the value of standard error, but also giving a truer representation of the mean value across the spectrum.

3 **Supplementary Note 3**: Time-dependent valence occupancy

Supplementary Figure 5 shows the average 3*d* occupancy over the full range of MC-MC simulations, covering up to 1 picosecond. At time = 1 attosecond, both compounds retain valence occupations near that of the ground state. As time progresses, variations in their respective ensemble of Auger-Meitner cascades reduce the difference in valence occupation.

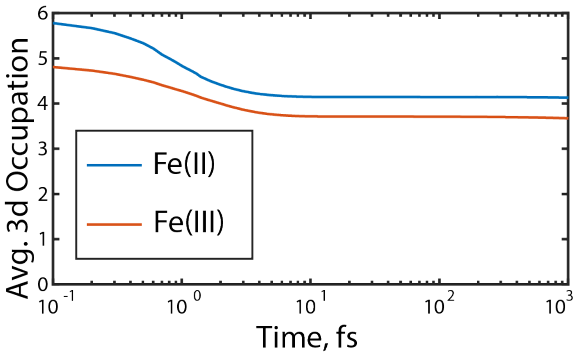

Supplementary Figure 5. Average 3*d* occupancy for both initial oxidation states as determined by the Monte Carlo simulations for all [Ne] $3s^2$ $3p^5$ states.

4 **Supplementary Note 4:** Additional X-ray spectra

Another method for performing this experiment would be to physically chop the pump pulse. As the character of the pump and probe are linked through the method used to produce both pulses, simply eliminating the pump undulators would alter the probe pulse, making comparisons ineffective. In this colinear geometry the option afforded the experimenter is to utilize the very absorption edge to be studied to block the pump. This can be achieved through the insertion of a thin foil of iron, although it does not provide an expeditious method for performing this experiment. Supplementary Figure 6 shows data of the Fe^{II} complex with 10 μm Fe foil in front of the sample area, interacting with both X-ray pulses. The attenuation length of Fe foil is 24.38 and 3.2 μm at 7.062 and 7.200 keV respectively, leading to transmitted fluence of 66.35% and 4.4%. Assuming the bilinear intensity relationship discussed in Supplementary Note 1 above, this corresponds to a 97% drop in signal strength seen as the elimination of both peaks.

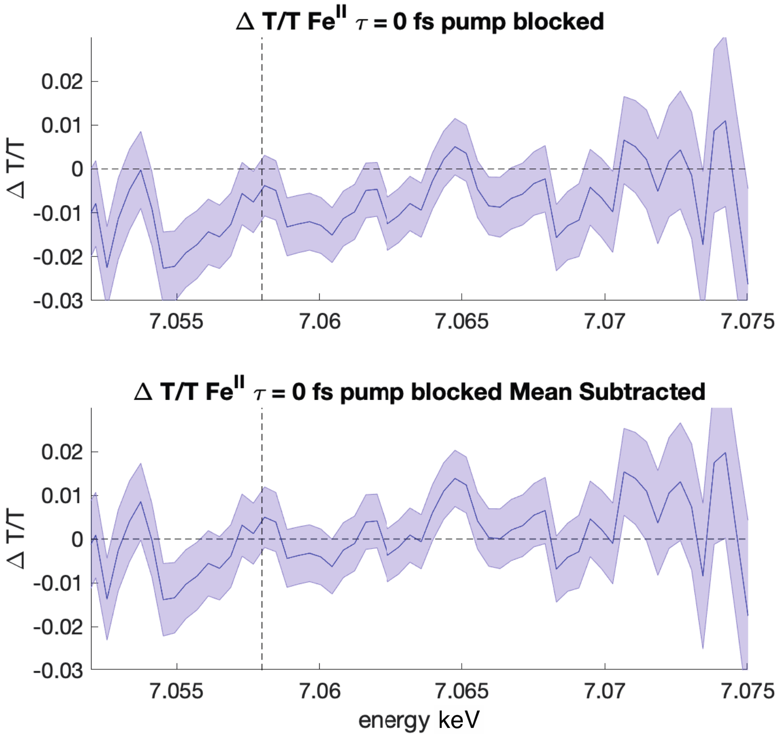

Supplementary Figure 6. Spectrum collected with 10μm Fe foil blocking beam path. This approximates a blocked pump pulse and results in the elimination of the signal. Vertical dashed lines represent the atomic Fe Kβ fluorescence line.

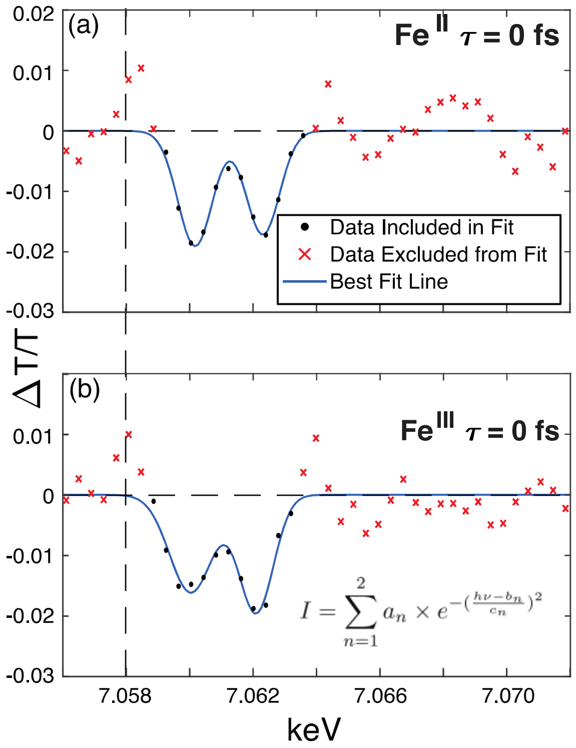

Supplementary Figure 7. Fits to the Gaussian peaks (equation inset) shown in Figure 3 of the main text. (a) Fit of the XPXP data for the Fe(II) complex and (b) fit of the XPXP data for the Fe(III) complex. The intensity, I, of the fitted data is dependent on the amplitude, a_n, central frequency, b_n, and width, c_n, of the two Gaussian peaks, where n=1,2. Red crosses show data omitted from the fitting routine. Vertical dashed lines represent the atomic Fe Kβ fluorescence line.

Supplementary Figure 7 shows complete fitting for the lines of best fit shown in Figure 3 of the main text. While fitting the two separate peaks as a sum of gaussians does indicate that the peaks shift between the two complexes, (~0.1 eV and ~0.2 eV respectively), the uncertainly in the fit limits the analysis based on this differentiation. Extracted fit parameters are provided in Supplementary Table 2. Uncertainties represented there are those of the fitting routine, not the experimental resolution. The pixel-to-pixel separation of the detector was ~0.4 eV.

Supplementary Table 2. Parameters and uncertainties of fitting routine for data in Figure 3

**Peak**	**Center Frequency [eV]**	**Amplitude [%]**	**Width (FWHM) [eV]**	**Relative Integrated Amplitude [arb. units]**
Fe^{II}	7,060.170±0.005	-1.908±0.150	1.966±0.010	0.957
Fe^{II}	7,062.310±0.005	-1.717±0.150	1.933±0.010	0.832
Fe^{III}	7,060.04±0.017	-1.615±0.310	2.185±0.329	1.000
Fe^{III}	7,062.09±0.013	-1.946±0.330	1.958±0.251	0.968

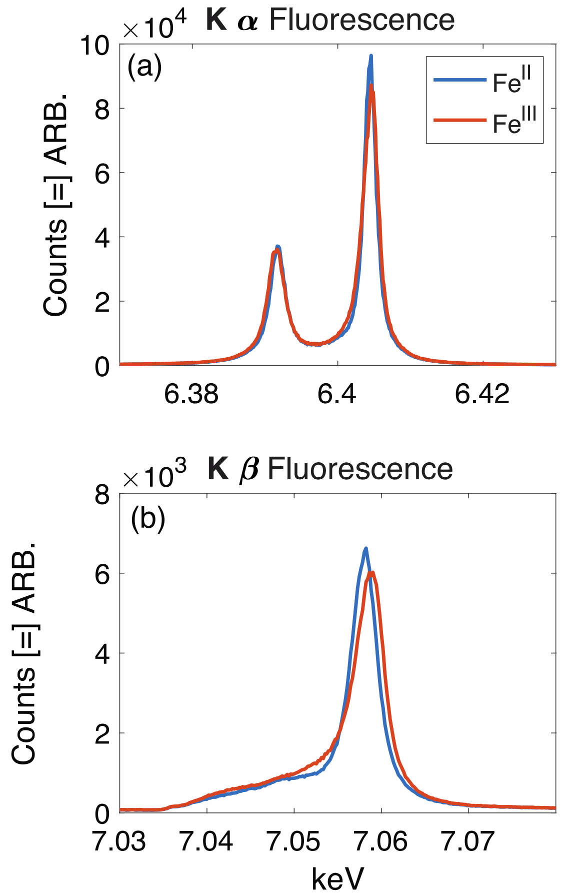

Supplementary Figure 8. Kα (a) and Kβ (b) Fluorescence data for Fe^{II} and Fe^{III} model complexes, displayed in counts. Peaks (at highest) intensity in the order [Kα 1; Kα 2; Kβ] for Fe^{II} = [37,120; 96,447; 6,626], Fe^{III} = [36,097; 87,230; 6,022]. The energy for Kβ is 7.05824 keV and 7.05884 keV for Fe^{II} and Fe^{III}, respectively. The ratio of total intensity Kα/Kβ is 13.7 and 13.5 respectively. Counts, in arbitrary units, match count units displayed in the Kβ RIXS spectra shown in Supplementary Figure 9. Details of data collection can be found in previously published work.[2-3]

Supplementary Figure 8 shows fluorescence data of both complexes collected at the Advanced Photon Source (APS) in 2015 as a reference for the reader. Additionally, we compare the published fluorescence yield values of the Fe atom with the probabilities obtained from the cascade simulation. Krause determined the fluorescence yield for iron to be ≈30%,[4] with relative intensities of Kα1:Kα2:Kβ to be 50:100:17 resulting in an estimated population of the Fe^{III} [Ne]$3s^23p^53d^5$ state to be approximately 3%, which is consistent with the simulation value of 4% in Figure 2 of the main text. Evidently, the [Ne]$3s^23p^53d^6$ state of the Fe^{II} complex is shorter lived. We note that this comparison relies on the approximation that the only channel for producing [Fe^{II} $3p^53d^6$] or [Fe^{III} $3p^53d^5$] states is through Kβ fluorescence and ignores the pathways of Kα fluorescence followed by L-edge fluorescence or corresponding Auger-Meitner processes.

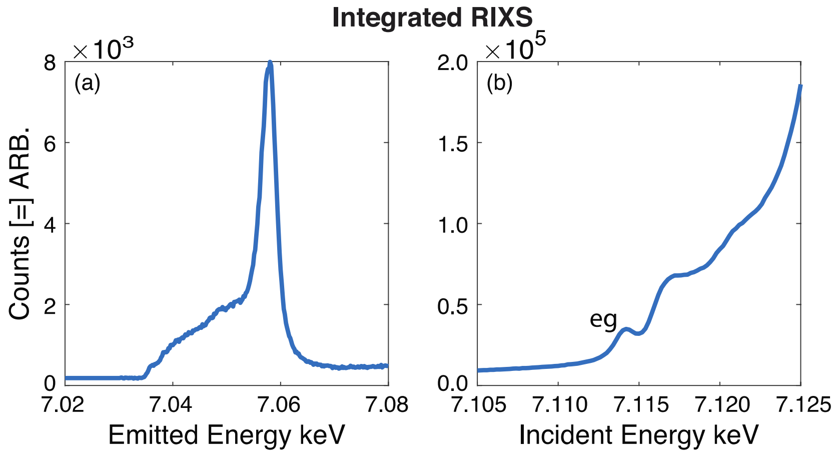

Supplementary Figure 9. Integrated Kβ RIXS spectra of 500 mM $K_4Fe^{II}(CN)_6$ in H_2O (reproducing the concentration used here) resolving the emission (a) and incident (b) energy axes. Count units are arbitrary, but consistent between the two plots. Details of data collection can be found in previously published work.[3]

Supplementary Figure 9 shows the integrated Kβ RIXS spectrum of the Fe^{II} complex in water. (a) shows the Kβ fluorescence channel intensity as an emission. (b) shows the XANES absorption resolved through the Kβ RIXS process. The intensity of the e_g peak in Supplementary Figure 9b and Kβ fluorescence in Supplementary Figure 8b are used as the ratio of intensities to plot the data in Figure 1 of the main text. Additionally, the transient 3*p* absorption intensity in Figure 1 of the main text is scaled so that the integral of a single 1*s*→3*p* root is equivalent to 1/6th the integrated Kβ fluorescence. From calculations, the relative oscillator strengths of the 1*s*→3*p* transitions are more than three orders of magnitude stronger than those of the 1*s*→3*d* transitions.

5 **Supplementary Note 5**: Computational details

5.1 Electron cascade simulations

In this on-the-fly Monte Carlo rate equation method,[5-6] all electronic processes are treated as random quantum processes. The probabilities are weighted by the transition rates and rates are computed using the Hartree-Fock-Slater (HFS) electronic structure method, which includes relativistic corrections and spin-orbit coupling terms, following a previously established procedure.[7] Following is simply the application of our previous simulations of Fe^{II},[8] to include both Fe^{II} and Fe^{III} oxidation states.

We performed 100,000 trajectories on the Fe^{2+} and Fe^{3+} ions in the low-spin configuration. Each cascade dynamics trajectory was initiated via the creation of a 1*s*-hole before the rest of the Auger-Meitner and fluorescence decay channels were computed. In each time step the probability of an electronic transition was computed and compared to a randomly generated number to determine the step's progression. Upon failure to execute a transition, time was advanced, while the system was held constant and compared to a newly generated random number. Successful transition steps made selections from available Auger-Meitner and fluorescence channels, weighted by their relative rates, and new rates were computed. From the time history of these transitions, the temporal profile of all participating electronic configurations were computed.

5.2 Electron structure calculations

All DFT and TDDFT-based XANES calculations were performed with the NWChem computational chemistry program.[9-11] The PBE0 exchange-correlation functional[12] was used as we have previously shown it to provide sufficient functionality in this case.[13-14] The COSMO (COnductor-like Screening MOdel)[15-16] implicit solvation model with a dielectric constant of 80.1 was used to represent the water solvent instead of an explicit solvent representation to reduce the complexity of the model. All electron calculations were performed using the 6-311G** basis set[17-18] for the light atoms and the Sapporo-TZP-2012 basis for the Fe atom.[19] The converged geometries of the ground state $[Fe^{II}(CN)_6]^{4-}$ and $[Fe^{III}(CN)_6]^{3-}$ molecular complexes are shown in Supplementary Table 3 and Supplementary Table 4, respectively. The reference hole configurations are generated by converging the Kohn–Sham orbitals with specified molecular orbital occupancies using the "occup" block feature in NWChem, which are then used for subsequent XANES calculations. By using TDDFT, we acknowledge that the spectra can only be captured within the space of single excitations. However, we believe this approach can provide a first order estimate if combined with additional supporting computations.

Supplementary Table 3. Geometry of the ground state $[Fe^{II}(CN)_6]^{4-}$ complex

Atom	X	Y	Z
Fe	0.00000000	0.00000000	0.00000000
C	-1.81917027	-0.03335831	-0.71926660
N	-2.91104648	-0.05028038	-1.14336000
C	-0.26363647	1.81748256	0.66107639
N	-0.39650765	2.91674740	1.04361053
C	-0.65245477	-0.72431645	1.71356864
N	-1.01949658	-1.14265108	2.74378932
C	0.26492194	-1.81715092	-0.66122605
N	0.39946334	-2.91608401	-1.04407649
C	0.65377106	0.72497185	-1.71283131
N	1.02222110	1.14453278	-2.74209377
C	1.81942656	0.03289085	0.71938992
N	2.91151676	0.04957108	1.14269602

Supplementary Table 4. Geometry of the ground state $[Fe^{III}(CN)_6]^{3-}$ complex

Atom	X	Y	Z
Fe	0.00000000	0.00000000	0.00000000
C	1.66434078	0.87659818	-0.59439314
N	2.65503328	1.38456560	-0.93290973
C	0.86003876	-0.55780875	1.68189365
N	1.37242765	-0.88008464	2.67574262
C	-0.47522468	1.68896229	0.89877624
N	-0.75605374	2.69018114	1.42127310
C	-0.86020289	0.55931993	-1.68139135
N	-1.37281553	0.88364520	-2.67454402
C	0.47285518	-1.68909585	-0.89889251
N	0.75091419	-2.69077309	-1.42192322
C	-1.66433206	-0.87600171	0.59530378
N	-2.65485687	-1.38303558	0.93556623

Supplementary Table 5. List of excited state roots represented in main text. Roots are scaled in plot by corresponding hole density.

Geometry	Configuration	Energy [eV]	Osc. Strength E-3
Fe^{II}	$3p^5\ t_{2g}^6$	7058.036	8.762
	$3p^5\ t_{2g}^5$	7060.150	8.755
	$3p^5\ t_{2g}^4$	7061.816	9.574
	$3p^4\ t_{2g}^6$	7064.089	8.656
	$3p^4\ t_{2g}^6$	7064.101	8.696
	$3p^3\ t_{2g}^5$	7069.212	8.684 x 3
Fe^{III}	$3p^5\ t_{2g}^5$	7060.121	8.698
	$3p^5\ t_{2g}^4$	7061.286	8.733
	$3p^4\ t_{2g}^3$	7064.281	8.674
	$3p^4\ t_{2g}^3$	7064.293	8.696
	$3p^3\ t_{2g}^4$	7068.109	8.676 x 1.5
	$3p^3\ t_{2g}^4$	7069.264	8.566 x 1.5

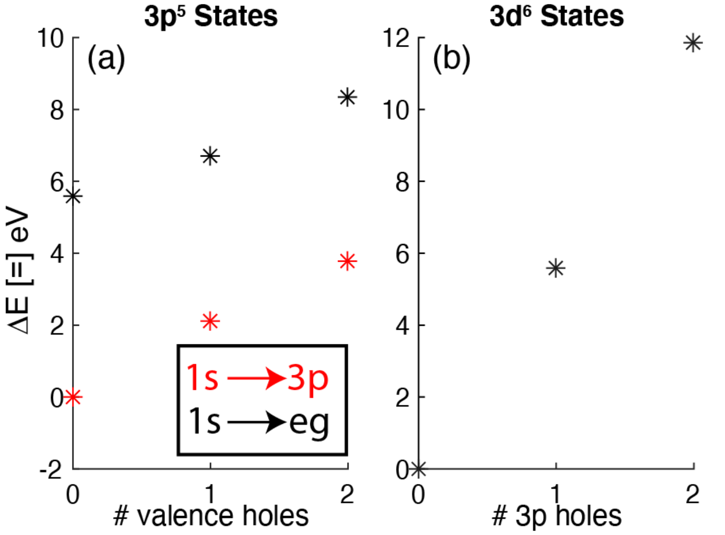

Supplementary Figure 10. Relative shifts for $Fe^{II}(CN)_6^{4-}$ of the 1*s*⟶3*p* and 1*s*⟶e_g transitions (K-edge) for (a) $3p^5$ states with varied number of valence holes and (b) $3d^6$ states with variable 3*p* holes. 1*s*⟶e_g transitions are plotted relative to the calculated transition energy for the $3p^63d^6$ configuration and showing the ~6 eV shift in e_g transition energy resulting from the 3*p* hole. 1*s*⟶3*p* energies are plotted relative to the calculated $3p^53d^6$ absorption energy. Linear fits are outlined below.

Supplementary Figure 10 shows the general trends in transition energies, in the Fe^{II} complex, responding to vacancies in either the 3*p* or the valence shells. Red stars, in (a), show that for each additional hole in the valence, the 1*s*→3*p* transition energy increases by approximately 2 eV. The

edge, represented by the 1s→e_g transition also shifts, starting by ~6 eV due to the 3p^5 configuration, but less significantly for each additional e_g hole. Supplementary Figure 10b shows that the edge shifts by ~6 eV for each additional hole in the 3p. This is what leads to the significant gap between 3p^4 and 3p^5 states. Comparing the slope of each linear fit describes the extent of 3p-3d interactions. Linear fits for Fe^{III} would only cover the 3d^5 and 3d^4 configurations, but their difference in energy can be used analogously, and are marked with (*) in Supplementary Table 6. We note that any initial shift in dipole energy relative to the experimental Kβ emission energy is dependent on the applied energetic shift. A comparison independent of that shift would directly compare the energy difference between calculated 1s→3p^5 peaks.

Supplementary Table 6. Fitting of transition energies as a function of hole density

Compound	Transition	Changing # holes	Slope [=] eV/hole	Intercept [=] eV	R^2
Fe^{II}	1s→3p	e_g	1.891	0.0747	0.9953
Fe^{II}	1s→e_g	e_g	1.377	5.503	0.9884
Fe^{II}	1s→eg	3p	5.929	-0.1132	0.9989
Fe^{III}	1s→3p	e_g	1.165*	---	---
Fe^{III}	1s→e_g	e_g	3.619*	---	---

Supplementary References

1. Liang, M.; et al. The Coherent X-ray Imaging instrument at the Linac Coherent Light Source. *J. Synchrot. Radiat.* **2015,** *22* (3), 514-519.
2. Biasin, E.; et al. Direct observation of coherent femtosecond solvent reorganization coupled to intramolecular electron transfer. *Nature Chemistry* **2021,** *13* (4), 343-349.
3. Liekhus-Schmaltz, C.; et al. Femtosecond X-ray Spectroscopy Directly Quantifies Transient Excited-State Mixed Valency. *The Journal of Physical Chemistry Letters* **2022**, 378-386.
4. Krause, M. O. Atomic radiative and radiationless yields for K and L shells. *Journal of Physical and Chemical Reference Data* **1979,** *8* (2), 307-327.

5. Ho, P. J.; Bostedt, C.; Schorb, S.; Young, L. Theoretical Tracking of Resonance-Enhanced Multiple Ionization Pathways in X-ray Free-Electron Laser Pulses. *Physical Review Letters* **2014,** *113* (25), 253001.
6. Ho, P. J.; Kanter, E. P.; Young, L. Resonance-mediated atomic ionization dynamics induced by ultraintense x-ray pulses. *Phys Rev A* **2015,** *92* (6), 063430 DOI: 10.1103/PhysRevA.92.063430.
7. Herman, F.; Skillman, S. *Atomic structure calculations*. Prentice-Hall: Englewood Cliffs, N.J, 1963.
8. Liekhus-Schmaltz, et al. Ultrafast x-ray pump x-ray probe transient absorption spectroscopy: A computational study and proposed experiment probing core-valence electronic correlations in solvated complexes. *J. Chem. Phys.* **2021,** *154* (21), 214107.
9. Valiev, M.; et al. NWChem: A comprehensive and scalable open-source solution for large scale molecular simulations. *Comput Phys Commun* **2010,** *181* (9), 1477-1489.
10. Aprà, E.; et al. NWChem: Past, present, and future. *The Journal of chemical physics* **2020,** *152* (18), 184102.
11. Lopata, K.; Van Kuiken, B. E.; Khalil, M.; Govind, N. Linear-Response and Real-Time Time-Dependent Density Functional Theory Studies of Core-Level Near-Edge X-Ray Absorption. *Journal of Chemical Theory and Computation* **2012,** *8* (9), 3284-3292.
12. Adamo, C.; Barone, V. Toward reliable density functional methods without adjustable parameters: the PBE0 model. *J. Chem. Phys.* **1999,** *110* (13), 6158-6170.
13. Ross, M.; et al. *The journal of physical chemistry. B* **2018,** *122* (19), 5075-5086.
14. March, A. M.; et al. Elucidation of the photoaquation reaction mechanism in ferrous hexacyanide using synchrotron x-rays with sub-pulse-duration sensitivity. *The Journal of chemical physics* **2019,** *151* (14), 144306.

15. Klamt, A.; Schueuermann, G. COSMO: a new approach to dielectric screening in solvents with explicit expressions for the screening energy and its gradient. *Journal of the Chemical Society, Perkin Transactions 2: Physical Organic Chemistry (1972-1999)* **1993,** (5), 799-805.

16. York, D. M.; Karplus, M. A Smooth Solvation Potential Based on the Conductor-Like Screening Model. *The Journal of Physical Chemistry A* **1999,** *103* (50), 11060-11079.

17. Krishnan, R.; Binkley, J. S.; Seeger, R.; Pople, J. A. Self-consistent Molecular Orbital Methods. XX. A Basis Set for Correlated Wave Functions. *J. Chem. Phys.* **1980,** *72*, 650-654.

18. McLean, A. D.; Chandler, G. S. Contracted Gaussian Basis Sets for Molecular Calculations. I. Second Row Atoms, Z=11-18. *J. Chem. Phys.* **1980,** *72*, 5639-5648.

19. Noro, T.; Sekiya, M.; Koga, T. Segmented contracted basis sets for atoms H through Xe: Sapporo-(DK)-nZP sets (n=D, T, Q). *Theoretical Chemistry Accounts* **2012,** *131* (2), 1124.
